# PET imaging utility of a novel Aβ-tracking PET radiotracer, [^18^F]FC119S in aged vervet monkeys

**DOI:** 10.1186/s12967-025-07642-5

**Published:** 2026-01-08

**Authors:** Bhuvanachandra Bhoopal, Brett M. Frye, Mack Miller, Avinash Bansode, Krishna K. Gollapelli, Richard A. Barcus, Samuel N. Lockhart, Naresh Damuka, Courtney L. Sutphen, Ryan W. Fitzgerald, Jeongchul Kim, Mark G. Baxter, Matthew J. Jorgensen, Suzanne Craft, Thomas C. Register, Christopher T. Whitlow, Carol A. Shively, Kiran K. Solingapuram Sai

**Affiliations:** 1 Department of Radiology, Wake Forest University School of Medicine, Winston-Salem, NC 27157 USA; 2 Department of Pathology, Section on Comparative Medicine, Wake Forest University School of Medicine, Winston-Salem, NC USA; 3 Department of Internal Medicine, Wake Forest University School of Medicine, Winston-Salem, NC USA

**Keywords:** Nonhuman primates, Radiotracer, PET imaging, Biomarkers, Aged vervets

## Abstract

**Background:**

Alzheimer’s disease is characterized by the deposition of amyloid-beta (Aβ) plaques, necessitating early detection and reliable biomarkers for effective intervention. Non-human primates, particularly aged vervet monkeys, offer valuable models for studying age-related Aβ pathology due to their close phylogenetic relationship to humans and similar neuropathological features.

**Methods:**

This study assessed the utility of [^18^F]FC119S, a novel Aβ-targeting PET radiotracer, in aged vervet monkeys. The radiochemistry of [^18^F]FC119S was optimized and automated to ensure high radiochemical purity and molar activity. PET/MRI imaging was performed to evaluate tracer uptake, distribution, and washout kinetics. Correlations between [^18^F]FC119S uptake and cerebrospinal fluid (CSF) and plasma biomarkers—including Aβ_42/40_ ratio, pTau181, neurofilament light chain (NfL), and pTau181/Aβ_42_ ratio—were analyzed. Autoradiography was conducted to validate regional tracer binding in brain tissues.

**Results:**

[^18^F]FC119S demonstrated high brain uptake, rapid washout, and widespread cortical distribution in vervet monkeys, mirroring patterns observed in human studies. Tracer uptake showed negative associations with CSF Aβ_42/40_ ratio in Aβ-affected regions, and significant positive correlations with CSF pTau181 and CSF pTau181/Aβ_42_ ratio in the temporal lobe. Additionally, significant positive correlations were observed between [^18^F]FC119S uptake and CSF NfL in the anterior cingulate gyrus, parietal, and occipital lobes. Autoradiography confirmed elevated tracer binding in specific brain regions of older vervets with low CSF Aβ_42_ compared to younger counterparts.

**Conclusions:**

These findings validate [^18^F]FC119S as a promising PET radiotracer for tracking Aβ deposition in aged vervet monkeys. Its imaging characteristics and biomarker correlations support its translational potential for Alzheimer’s disease research and early diagnostic applications.

**Supplementary Information:**

The online version contains supplementary material available at 10.1186/s12967-025-07642-5.

## Introduction

Non-human primates (NHP) are valuable models of brain aging as they are similar to humans in neurobiology and anatomy, metabolism, and pharmacokinetics [1]. Aged vervet monkeys (*Chlorocebus aethiops sabaeus*, also known as African green monkeys) are excellent models of age-associated beta-amyloid (Aβ) deposition due to their phylogenetic proximity to humans. Their Aβ peptide, a hallmark pathology of Alzheimer’s disease (AD), has 100% sequence homology with human Aβ and naturally accumulates in the brain with age [2, 3]. We have documented age-related early AD–like neuropathologic changes and their associations with cerebrospinal fluid (CSF) biomarkers, brain structure (assessed via MRI), [^18^F]-Fluorodeoxyglucose PET, and behavior in older vervet monkeys [4]. Similar to AD pathogenesis in humans, increased Aβ and phospho-tau burden were associated with reduced brain volume, diminished glucose metabolism, and impaired complex behaviors such as gait speed [4, 5]. In addition, the distribution of Aβ plaques across the cerebral cortex mirrors that observed in early AD pathology in humans [6, 7], underscoring the value of aged vervets for exploring early Aβ-related mechanisms and for testing novel therapeutic strategies [4].

A panel of hyperphosphorylated peptides forms a ubiquitous component of cerebral and visceral Aβ deposits; studying the changes in these deposits can facilitate AD patient prognosis and treatment response [8]. Within the brain, these Aβ deposits can form either high (nM) or low (µM) affinity Aβ fibril binding sites [9]. Although both sites provide critical biochemical information through neuroimaging, existing PET radioligands predominantly occupy high-affinity sites [9, 10]. For example, binding studies with [^11^C]PiB in human AD brain fibrils indicate a majority of the signal arising from high-affinity sites [11]. Consequently, [^11^C]PiB uptake in low-affinity Aβ binding sites of the cortex is minimal, resulting in lower cortex: cerebellum (target: non-target) with an uptake ratio of < 2, with significant uptake in white matter. In transgenic AD mice, detecting the sparse, low-affinity sites would hypothetically require a ten-fold increase in molar activity [12, 13].

In contrast, aged NHP brains (including squirrel monkeys, vervets, macaques, and chimpanzees) possess abundant low-affinity Aβ binding sites despite similar levels of the Aβ peptide [9]. While the exact mechanistic role of these low-affinity binding sites in AD progression remains unknown [9], their abundance in aged vervets with early AD pathology [4, 6, 7] offers a unique opportunity to evaluate imaging properties of new Aβ-targeted PET radiotracers. Real-time in vivo imaging of these sites with a sensitive PET ligand can elucidate their role in AD pathogenesis. Therefore, there is a need for PET radioligands with optimized in vivo uptake kinetics, more importantly, enhanced sensitivity to low-affinity Aβ sites in vervet monkeys. In particular, [^18^F]-labeled tracers are preferable due to their longer half-life, which facilitates broader application and improved imaging protocols.

A novel Aβ-imaging PET radiotracer, [^18^F]FC119S was reported to have high binding affinity for the Aβ_1–42_ protein aggregate (0.16 nM) and AD brain homogenate (13–15 nM) with a distinct excretion pattern from the frontal cortex [14]. In a pilot study, [^18^F]FC119S showed significantly higher cortical uptake in AD patients compared to healthy controls [15]. Additionally, in healthy rhesus monkeys, [^18^F]FC119S demonstrated high brain uptake, rapid washout, and widespread distribution across all cortical regions [16]. Recent advances in Aβ PET imaging have highlighted limitations in widely used radioligands such as [^11^C]PiB and [^18^F]Amyvid, including suboptimal specificity and pharmacokinetic properties that hinder their effectiveness in aged NHP models of AD [17]. In contrast, [^18^F]FC119S has demonstrated superior performance over these existing radioligands, characterized by high-target tissue binding, favorable brain kinetics, and a robust safety profile [15]. These features suggest that [^18^F]FC119S may offer enhanced sensitivity and reliability for detecting Aβ pathology in aged NHPs. However, its efficacy in aged NHP models remains to be systematically evaluated. In this study, the radiochemistry of [^18^F]FC119S was automated and its regional brain uptake in aged vervet monkeys was evaluated using PET imaging and autoradiography. Furthermore, radiotracer brain uptake was correlated with various CSF and plasma biomarkers to assess its utility in aged vervets, representing a critical step toward validating its translational potential for AD research.

## Methods

### Radiochemistry

**Scheme 1:**
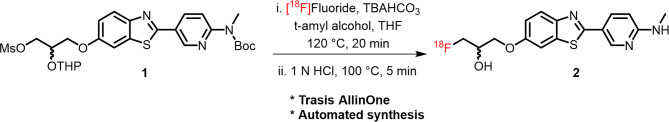
Automated synthesis of [^18^F]FC119S

Radiochemistry of [^18^F]FC119S was performed in an TRASIS-AIO module by automated synthesis, using the reported methodology with minor modifications (Scheme 1) [16]. The [^18^F]F^−^ ion, generated by irradiating [^18^O]H_2_O with a cyclotron (GE PETtrace), was captured using a Chromafix^®^ (PS-HCO_3_) cartridge and eluted into the reaction vial with a methanol solution (0.8 mL) containing tetra-n-butylammonium bicarbonate (TBAHCO_3_, 5 mg) and water (40 µL). After removing the solvent through azeotropic evaporation with acetonitrile (0.5 mL) at 100 °C under a nitrogen stream, a solution of the precursor (**1**) (5 mg) in tetrahydrofuran (50 µL) and t-amyl alcohol (1.0 mL) was added to the reaction vial. The reaction mixture was heated at 120 °C for 20 min, and the solvent was evaporated with nitrogen at 100 °C. Later 1 N HCl aqueous solution (0.9 mL) was added and stirred at 100 °C for 5 min. After cooling to 40 °C, the reaction mixture was neutralized with 1 N NaOH solution (0.9 mL) and loaded onto a C18 Sep-Pak cartridge (Waters, Lot number: 046333088 A). After washing with water (5 mL) and eluting with acetonitrile (2 mL) into a vial, it was injected into a semipreparative HPLC system (Luna^®^ 5 μm C18 100 Å LC Column 250 × 10 mm; mobile phase: isocratic, acetonitrile/water (50/50); flow rate: 3 mL/min; UV = 254 nm). Fraction containing [^18^F]FC119S was collected, diluted with water (15 mL), loaded onto an activated C18 Sep-Pak cartridge, eluted with ethanol and diluted with saline (10% ethanol in saline solution) to obtain the final product. Chemical and radiochemical purity were assessed using an analytical HPLC (Prodigy™ 5 μm ODS-3 100 Å, LC Column 250 × 4.6 mm) with a mobile phase of CH_3_CN/H_2_O (50/50, v/v) at UV = 254 nm and a flow rate of 0.5 mL/min.

### Animal housing

All vervets (*n* = 17) were middle to old-aged females (15–27 years old) housed in the Vervet Research Colony (VRC) at the Wake Forest Primate Research Center, living in stable family groups within large indoor-outdoor enclosures. All animal housing, handling, and experimental procedures were performed in accordance with the National Institutes of Health Guide for the Care and Use of Laboratory Animals and were approved by the Institutional Animal Care and Use Committee (IACUC) at Wake Forest University School of Medicine. Animal environmental enrichment followed IACUC’s Non-Human-Primate Environmental Enrichment Plan.

### CSF and plasma biomarkers quantification

CSF samples were taken by inserting a 22-gauge needle percutaneously into the cisternal space while the ketamine-sedated animal was maintained in a lateral recumbent position as previously described [18]. Approximately 1–1.5 cm^3^ of spinal fluid was obtained and frozen at -80℃ until analysis. CSF and plasma samples were taken twice a year to estimate the biomarker levels. Assessments of Aβ_42_, Aβ_40_, and pTau181 were conducted using first-thawed CSF or plasma with Mesoscale Discovery V-PLEX Plus Aβ Peptide Panel 1 (6E10) (Cat# K15200G) kits and S-PLEX NHP Tau (pTau181) kits (Cat# K156AGMS) as described by the manufacturer. CSF and plasma neurofilament light chain (NfL) were measured using an in-house duplex assay employing MSD R-PLEX antibody sets for NfL paired with U-PLEX Development Pack 2-Assay SECTOR plates with modifications to the MSD standard instructions. Mean coefficient of variation (CV) across all CSF samples was < 3% for Aβ_42_, Aβ_40_, and NfL, and 5.5% for pTau181. For plasma, CVs were < 3.1% for Aβ_42_ and Aβ_40_, < 2.4% for NfL, and < 5.1% for pTau181. All the above assays have been validated in vervet and macaque samples internally. Biomarker values from CSF and plasma samples were selected closest to PET scanning dates.

### MRI imaging protocol

On the day of scanning, all animals were transported from the VRC to the MRI Imaging Center at Wake Forest University School of Medicine. Upon arrival, animals were sedated with ketamine hydrochloride (10–15 mg/kg body weight, i.m.), followed by maintenance of anesthesia with isoflurane (3% for induction, 1.5% for maintenance) throughout the imaging procedure. MRI scans were acquired using a 3T Siemens Skyra scanner (Siemens Healthcare) with a 3D volumetric magnetization-prepared rapid gradient echo sequence (TR = 2700 ms; TE = 3.32 ms; TI = 880 ms; flip angle = 8°; 192 slices; voxel size = 0.5 × 0.5 × 0.5 mm³) [19].

### PET/CT imaging of [^18^F]FC119S in vervets

Dynamic 0–120 min PET/CT imaging of [^18^F]FC119S was performed on a GE Discovery PET/CT scanner [20]. All vervets were fasted overnight prior to scanning and on the day of scanning, animals were anesthetized with ketamine (10 mg/kg, i.m.) and transported to the PET Center at Wake Forest University School of Medicine. Isoflurane (3–5%) was administered via nose cone for induction, followed by endotracheal intubation and maintenance with 1.5% isoflurane in oxygen throughout the scan. [^18^F]FC119S (~ 0.37 GBq) was administered intravenously via a catheter placed in the external saphenous vein. The body temperature was maintained at ~ 40 °C using a water-circulating heating pad throughout the scanning session. Vitals including heart rate, blood pressure, respiratory rate, and body temperature, were continuously monitored [20].

### Image analysis

Atlas-based Volumes of Interest (VOIs) were defined using the INIA19 non-human primate brain atlas provided in PMOD. Regions included whole brain, gray matter, white matter, cortical regions (frontal, parietal, temporal, and occipital lobes), anterior and posterior cingulate gyrus, hippocampus, and cerebellum. Standard uptake values (SUVs) and time-activity curves (TACs) were calculated over the 0–120 min (5 min interval) using co-registered PET/MRI data using PMOD Biomedical Image Quantification and NEURO tools (version 4.3; PMOD Technologies LLC, Zurich, Switzerland) [20–22]. SUVs were normalized to cerebellar uptake to generate standardized uptake value ratios (SUVrs). Area under the curve (AUC) values were derived from TACs using the trapezoidal integration method in GraphPad Prism (version 10.1.2) to quantify total radiotracer uptake across brain regions. All analyses were conducted by investigators blinded to individual vervet information, including age, sex, body weight, and biomarker profiles, to minimize bias and ensure objective data interpretation. Blinding was maintained by assigning anonymized identification codes to each subject, with all metadata withheld during image processing. Only after completion of all analyses, the subject identifiers were decoded for interpretation and correlation with biological variables.

### Autoradiography studies

Autoradiography studies using [^18^F]FC119S were conducted on post-mortem brain sections of younger-adult vervets with high CSF Aβ_42_ levels (*n* = 2; ages 13.4 and 26.1 yrs) and older-adult vervets with low CSF Aβ_42_ levels (*n* = 2; ages 28.6 and 29.9 yrs) obtained from Wake Forest NHP brain pathology lab as previously described [20]. In brief, brain tissue sections were collected from multiple regions implicated in AD pathology, including prefrontal cortex, pons, hippocampus, frontal cortex, midbrain/thalamus/hypothalamus, and cerebellum. Tissue sections, 20 μm thick, were mounted on glass slides (Superfrost Plus slides, Fisher Scientific, Waltham, MA) following air-drying for 30 min. Nonradioactive FC119S (200 µM) was added to slides 30 min prior to radiotracer treatment for blocking experiments. Tissue slides were then pre-incubated in ice cold acetone for 2 min followed by washing in 1× PBS (3 × 5 min). All slides were incubated in a solution containing [^18^F]FC119S (37 KBq/1 mL) in 1× PBS for 1 h. The slides were washed in an order of 1 × PBS for 2 min, 70% EtOH/ 30% 1× PBS for 2 min, 30% EtOH/70% 1× PBS for 1 min, and finally in 1× PBS for 1 min and quickly air-dried. The radiotracer-treated brain tissue slides were exposed to a radioluminographic imaging cassette (BAS-IP SR 2025, GE Healthcare, Marlborough, MA) for 24 h at − 20 °C. The cassettes were then scanned with a GE Amersham Typhoon scanner (25 μm pixel size). Autoradiograms were analyzed using ImageQuant TL 8.2 software. Specific binding was calculated in PSL/mm^2^ from regions of interest which were drawn manually using MCID core 7.1 software as described earlier [23, 24].

### Statistical analysis

Statistical analyses were performed using GraphPad Prism (version 10.1.2; GraphPad Software, San Diego, CA). Correlation coefficients (r) were calculated to assess the strength and direction of associations between radiotracer uptake and variables such as age and fluid biomarker levels. Corresponding two-tailed p-values were computed to determine statistical significance, with a threshold of *p < 0.05 considered significant. Data were visually inspected for normality and outliers prior to analysis, and results were reported with appropriate measures of central tendency and variability.

## Results

### Radiochemistry of [^18^F]FC119S

[^18^F]FC119S synthesis was fully optimized and automated in the commercial TRASIS-AIO module (Scheme 1**)**. The total synthesis time was ~ 60 min, starting from [^18^F]F^-^ drying, radiolabeling, deprotection, semi-preparative HPLC purification, and final reformulation. Retention times of the product from semi-preparative and analytical HPLC columns were 7–8 min and 9–10 min, respectively. Product purity was authenticated by co-injecting [^18^F]FC119S with its non-radioactive standard on analytical HPLC, confirming similar retention time (9–10 min). [^18^F]FC119S was produced with radiochemical yield of ~ 12 ± 5% (decay-corrected to the end of synthesis), consistently achieving high reproducibility, high radiochemical purity (> 98%) and molar activity (~ 140 GBq/µmol) across more than 25 independent production runs.

### PET/MRI imaging

**Fig. 1:**
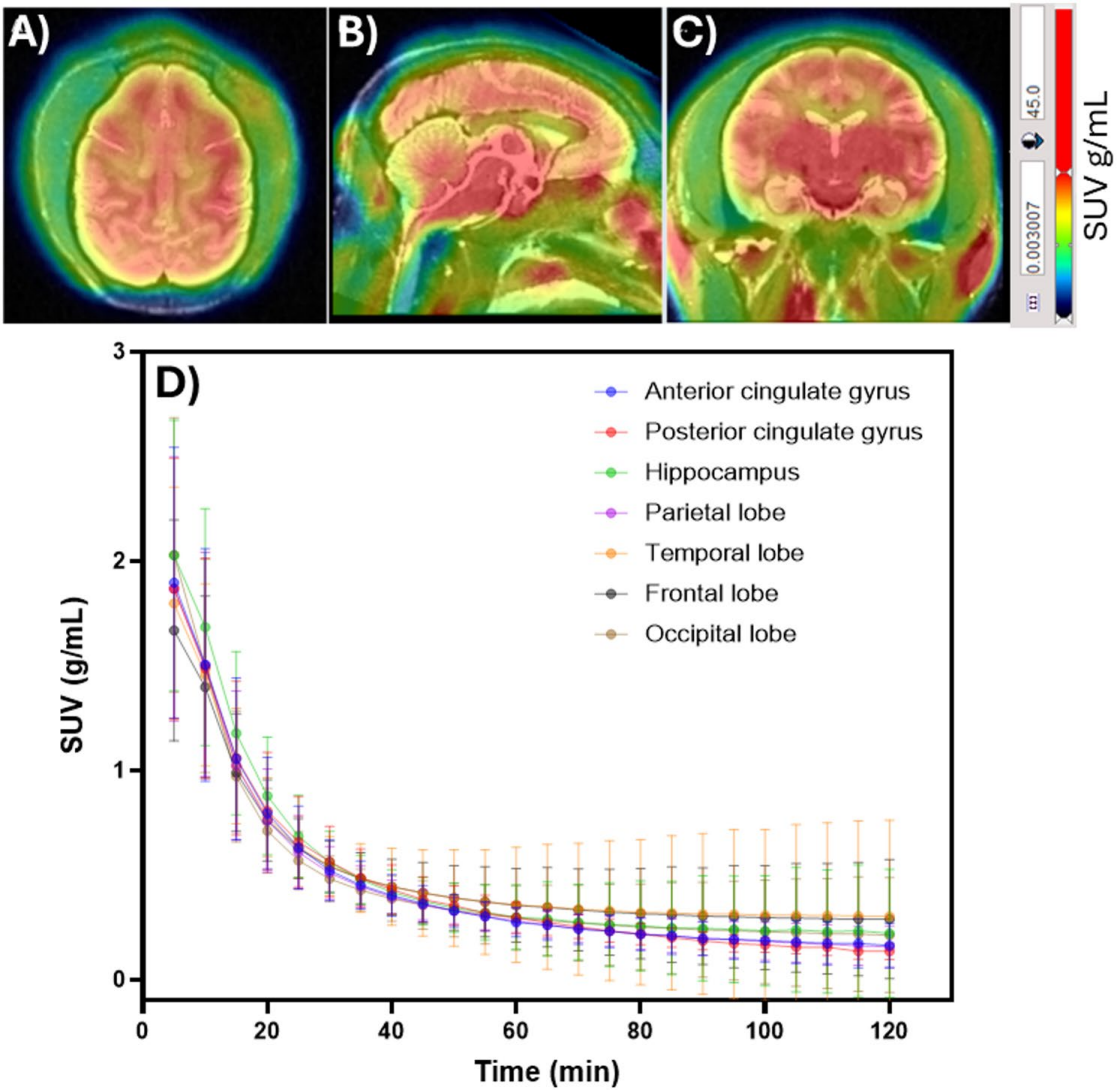
Representative summed (0–120 min) **A**) axial, **B**) sagittal, and **C**) coronal PET-MR images following an i.v. injection of [^18^F]FC119S in a vervet monkey (age: 24.1 yrs). **D)** Time-activity curve (TAC) analysis of [^18^F]FC119S in different brain regions over 120 min

All animals tolerated the imaging procedures well, with stable vital signs maintained throughout and no adverse events reported. Fused PET/MRI data confirmed the successful uptake of [^18^F]FC119S across multiple brain regions in vervet monkeys, with robust signal observed in both white and gray matter (Fig. 1A–C). SUVs and TACs were derived from the co-registered PET/MRI datasets. SUVr and AUC values were subsequently calculated from SUVs and TACs, respectively using trapezoidal integration to assess cumulative radiotracer uptake and used for quantitative analysis (Table S1 & S2). TAC profiles demonstrated acceptable pharmacokinetics i.e., rapid brain penetration of [^18^F]FC119S within 5–10 min post-injection, followed by a gradual washout over the 120-minute (Fig. 1D). SUVr and AUC metrics were further analyzed for correlations with age and fluid biomarker levels to establish the relationship between them (Table S3 & S4).

#### Correlations between tracer uptake and age

**Fig. 2:**
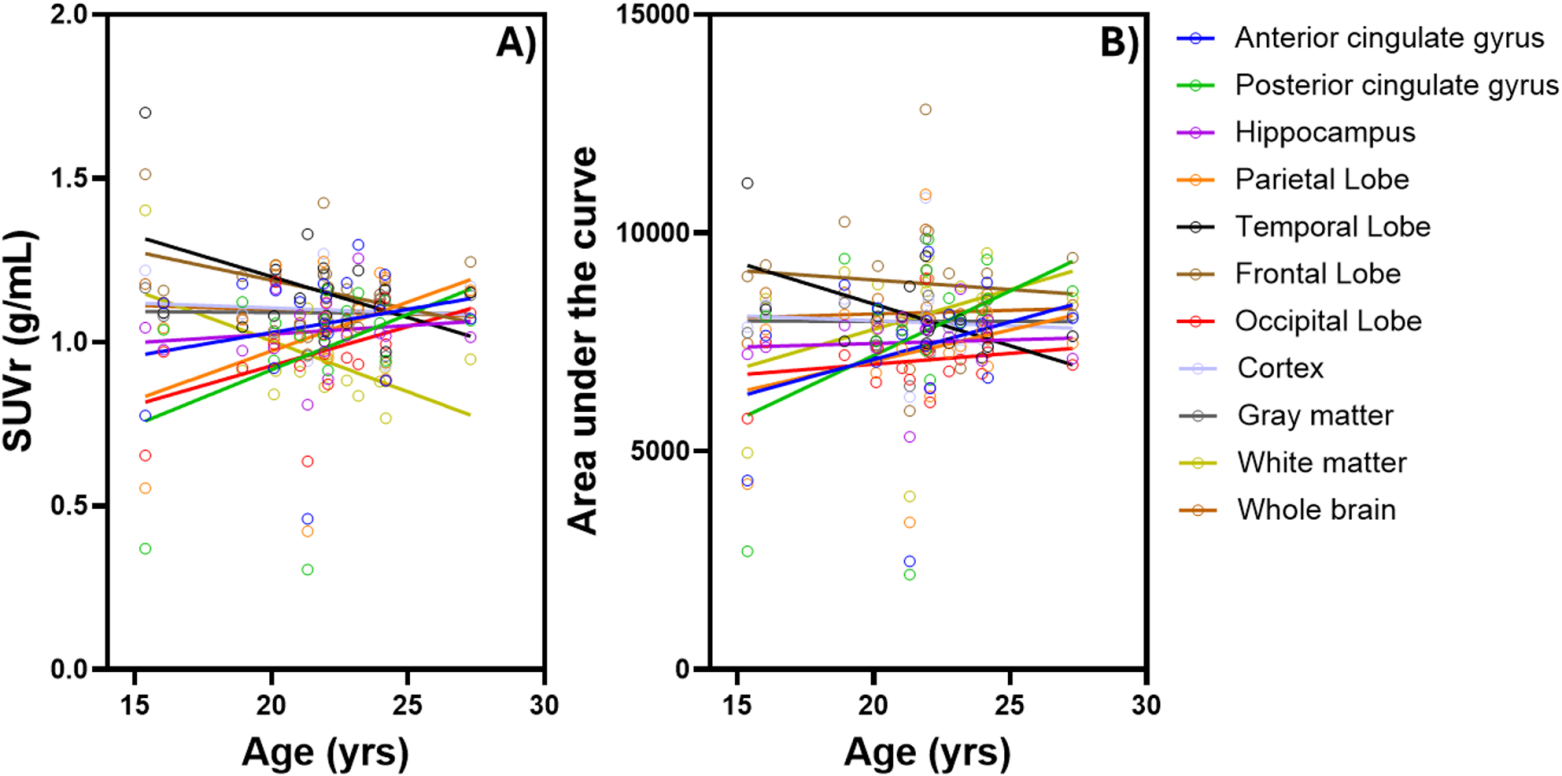
Correlations between age and [^18^F]FC119S PET **(A)** SUVr and **(B)** AUC in different brain regions

Despite the lack of statistical significance, positive trends were observed in the anterior and posterior cingulate gyri, hippocampus, parietal lobe, and occipital lobe (*r* = 0.14 to 0.40). In contrast, negative associations with age were noted in the temporal lobe, frontal lobe, white matter, and whole brain (*r* = − 0.10 to − 0.62), while cortex and gray matter exhibited slight negative trends (*r* = − 0.02 to − 0.09) (Fig. 2A). AUC values demonstrated a similar pattern to SUVr findings. Positive correlations with age were observed in the anterior and posterior cingulate gyri, parietal lobe, occipital lobe, and white matter (*r* = 0.21 to 0.40), with slight positive trends in the hippocampus and whole brain (*r* ≈ 0.06). The temporal lobe showed a negative correlation with age (*r* = -0.57), while the frontal lobe, cortex, and gray matter exhibited minimal negative associations (*r* = − 0.003 to − 0.08) (Fig. 2B).

#### Correlations between tracer uptake and fluid biomarkers

**Fig. 3:**
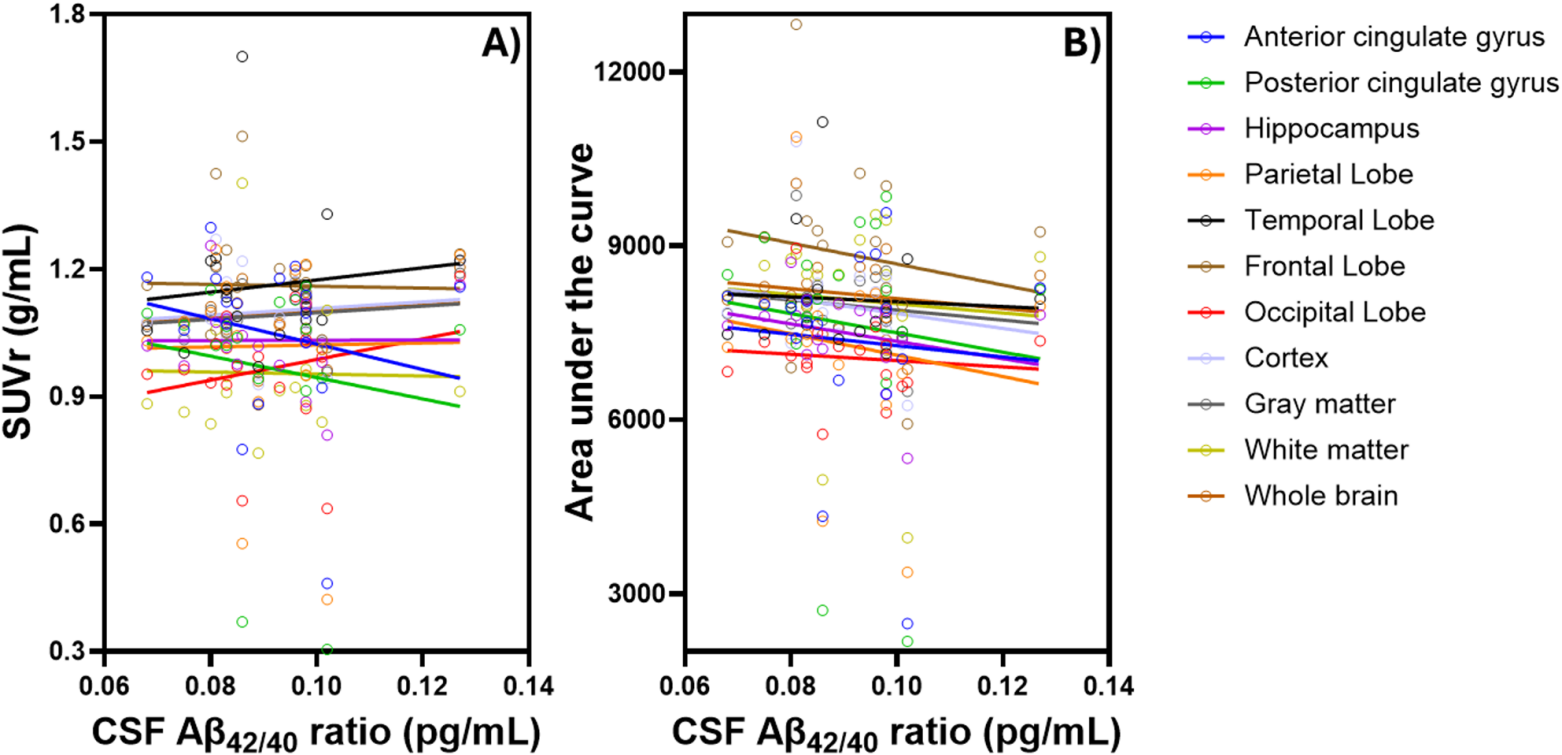
Correlations between Aβ_42/40_ ratio and [^18^F]FC119S PET **(A)** SUVr and **(B)** AUC in different brain regions

Correlation analyses revealed no statistically significant associations between SUVrs and the Aβ_42/40_ ratio across most brain regions. However, moderate negative correlations were observed in the anterior and posterior cingulate gyri (*r* = − 0.13 to − 0.20), with slight negative trends in the frontal lobe and white matter (*r* = − 0.01 to − 0.02). In contrast, moderate positive correlations were found in the temporal lobe, occipital lobe, cortex, gray matter, and whole brain (*r* = 0.11 to 0.22), while the hippocampus and parietal lobe showed minimal positive associations (*r* = 0.003 to 0.01) (Fig. 3A). AUC values demonstrated a similar overall pattern but with more consistent negative associations across regions. Moderate negative correlations were observed in the posterior cingulate gyrus, hippocampus, parietal lobe, frontal lobe, occipital lobe, cortex, gray matter, and whole brain (*r* = − 0.10 to − 0.26). Slight negative trends were also noted in the anterior cingulate gyrus, temporal lobe, and white matter (*r* = − 0.05 to − 0.07) (Fig. 3B).

**Fig. 4:**
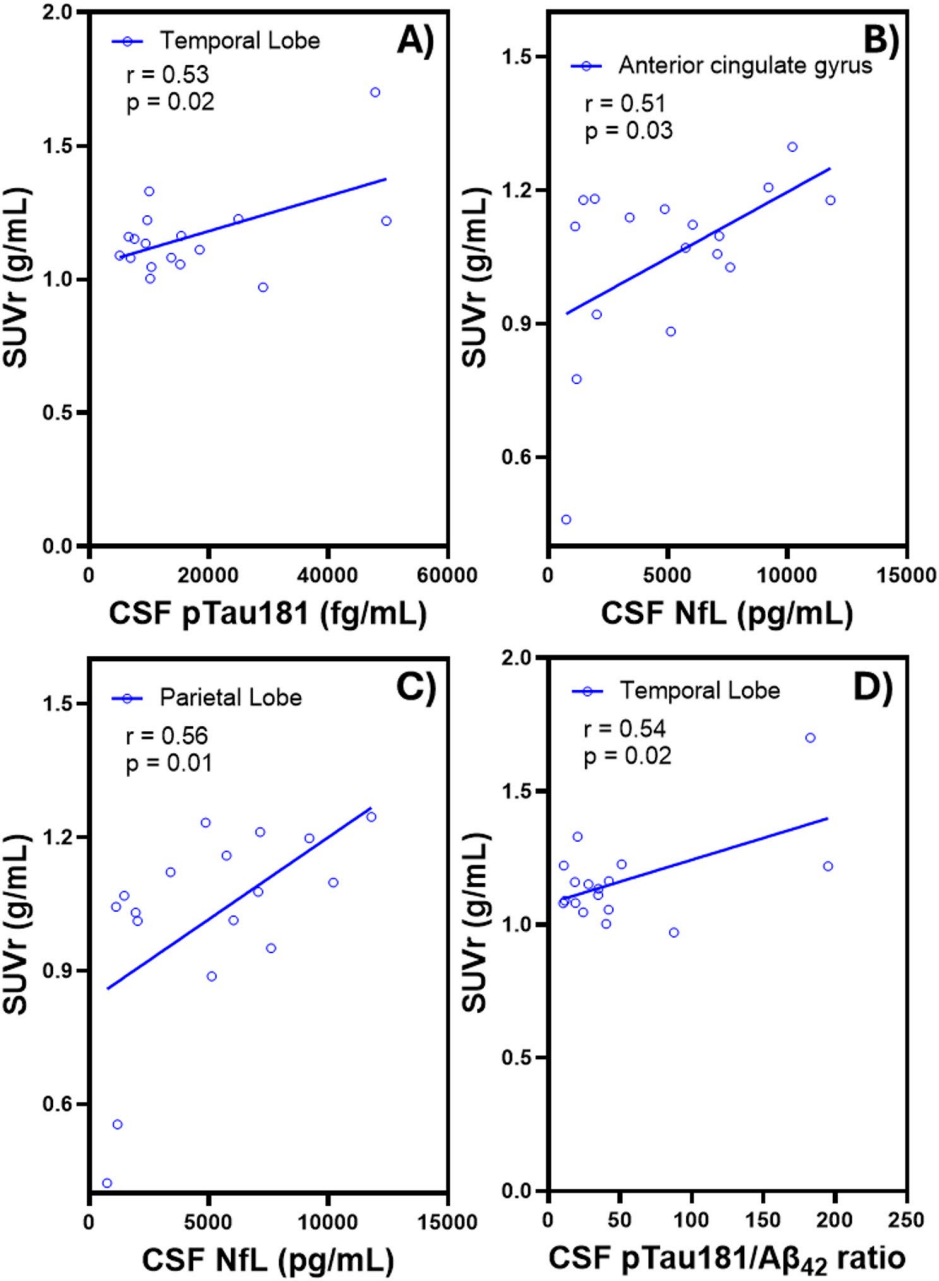
Significant correlations between [^18^F]FC119S PET SUVrs and **(A)** CSF pTau181 (*r* = 0.53, *p* = 0.02) in temporal lobe, **(B)** CSF NfL in anterior cingulate gyrus (*r* = 0.51, *p* = 0.03), **(C)** CSF NfL in parietal lobe (*r* = 0.56, *p* = 0.01), and **(D)** CSF pTau181/Aβ_42_ ratio in temporal lobe (*r* = 0.54, *p* = 0.02)

SUVr values in specific brain regions showed significant correlations with CSF biomarkers, including pTau181, NfL, and the pTau181/Aβ_42_ ratio. A significant positive correlation was observed between SUVr and CSF pTau181 levels in the temporal lobe (*r* = 0.53, *p* = 0.02) (Fig. 4A). Additionally, SUVr values were significantly associated in positive trend with CSF NfL levels in both the anterior cingulate gyrus (*r* = 0.51, *p* = 0.03) and the parietal lobe (*r* = 0.56, *p* = 0.01) (Fig. 4B-C). SUVr also demonstrated a significant positive correlation with the CSF pTau181/Aβ_42_ ratio in the temporal lobe (*r* = 0.54, *p* = 0.02) (Fig. 4D). No significant correlations were observed between SUVr and the plasma biomarkers.

**Fig. 5:**
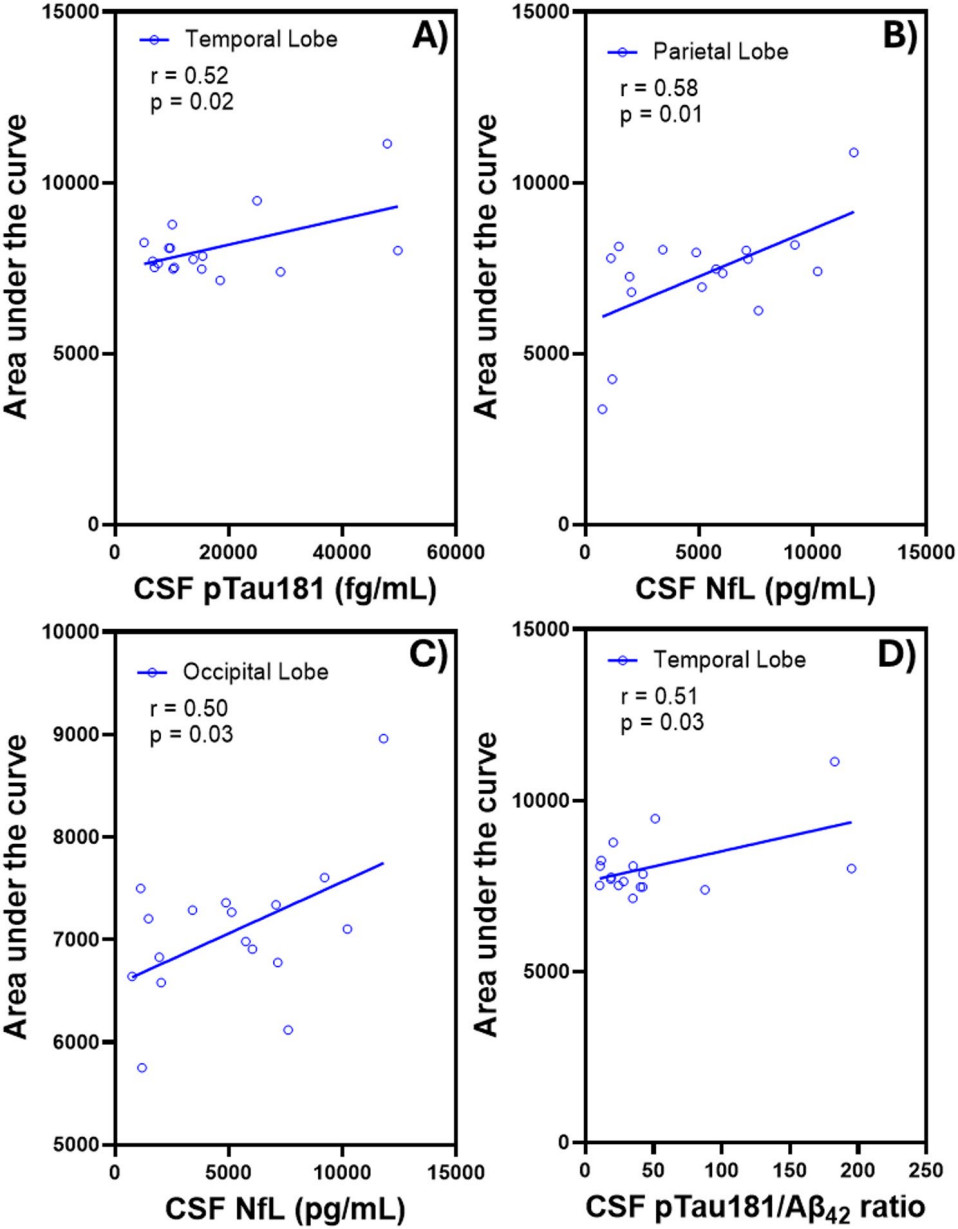
Significant correlations between [^18^F]FC119S PET AUC and **(A)** CSF pTau181 in temporal lobe (*r* = 0.52, *p* = 0.02), **(B)** CSF NfL in parietal lobe (*r* = 0.58, *p* = 0.01), **(C)** CSF NfL in occipital lobe (*r* = 0.50, *p* = 0.03), and **(D)** CSF pTau181/Aβ_42_ ratio in temporal lobe (*r* = 0.51, *p* = 0.03)

Consistent with SUVr findings, analysis of AUC values revealed significant region-specific correlations with CSF biomarkers, including pTau181, NfL, and the pTau181/Aβ_42_ ratio. In the temporal lobe, AUC values were significantly and positively correlated with CSF pTau181 levels (*r* = 0.52, *p* = 0.02) and with the CSF pTau181/Aβ_42_ ratio (*r* = 0.51, *p* = 0.03) (Fig. 5A, D). In addition, AUC values showed strong positive correlations with CSF NfL concentrations in the parietal lobe (*r* = 0.58, *p* = 0.01) and occipital lobe (*r* = 0.50, *p* = 0.03), indicating that increased tracer uptake in these regions may reflect underlying neurodegeneration (Fig. 5B–C).

### Autoradiography studies

**Fig. 6:**
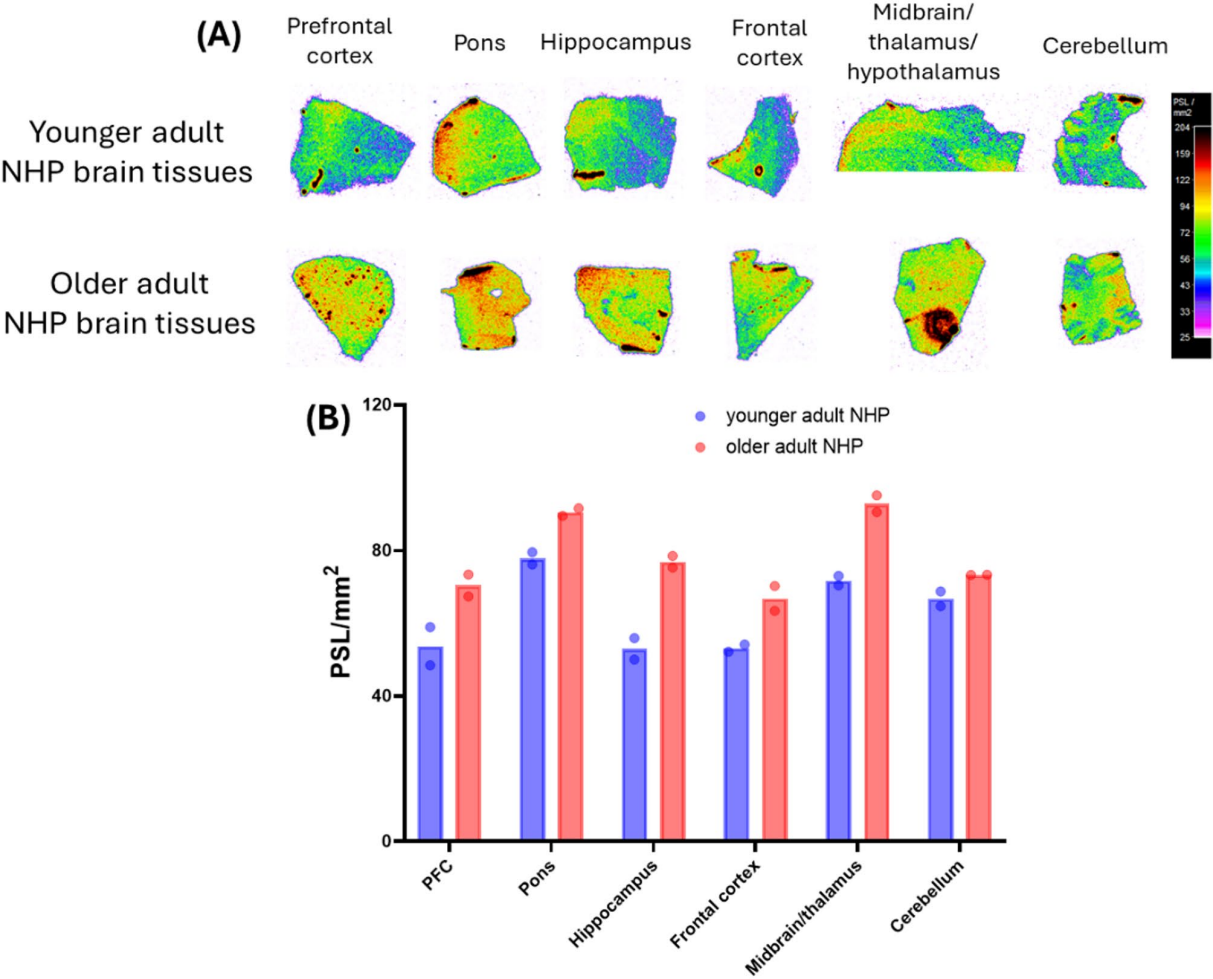
Autoradiograms of [^18^F]FC119S in different brain regions **(A)** and its regional quantification **(B)** in younger adult and older adult NHP brain tissues

Autoradiography analysis of post-mortem brain sections revealed higher [^18^F]FC119S uptake in all brain regions analyzed in older adult NHPs with low CSF Aβ₄₂ levels compared to younger adult NHPs with high CSF Aβ₄₂ levels (Fig. 6A). Quantitative comparisons across brain regions demonstrated region-specific increases in tracer binding in aged tissues. Specifically, [^18^F]FC119S uptake was 31% higher in the prefrontal cortex, 16% higher in the pons, 45% higher in the hippocampus, 25% higher in the frontal cortex, and 29% higher in the midbrain/thalamus/hypothalamus in older animals. In contrast, the cerebellum showed only a modest increase of 9% in tracer uptake (Fig. 6B).

## Discussion

AD is characterized by an extended preclinical stage preceding symptom onset, highlighting the need for early detection and diagnosis to enable timely and effective treatment. To address this, extensive research has focused on establishing reliable biomarkers for AD progression and early detection. Fluid biomarkers that are routinely studied for AD include amyloid peptides (Aβ_40_, Aβ_42_, and their ratio), total tau (t-Tau), phosphorylated tau (pTau), and NfL, measured in both CSF and plasma. Integrating advanced molecular imaging with neuropsychological assessments and fluid biomarkers is expected to enhance diagnostic accuracy, allowing the identification of early disease stages [25].

In the present study, regional [^18^F]FC119S uptake did not show a significant or strong associations with age across cortical and subcortical regions. Dividing vervets into younger (< 22 yrs) and older (> 22 yrs) groups also failed to show significant associations. These findings suggest that [^18^F]FC119S binding may be more closely linked to the presence of Aβ pathology than to chronological aging alone. Previous PET studies in both humans and NHPs have shown that while advancing age is associated with increased risk for Aβ deposition, the relationship between age and tracer uptake is often modest and highly region-dependent [4, 26]. In particular, age-related increases in cortical amyloid are more pronounced in individuals with underlying AD pathology, whereas cognitively normal aging brains often show heterogeneous or minimal tracer accumulation [27]. The absence of robust age effects in our cohort may therefore reflect a limited contribution of normal aging to [^18^F]FC119S binding, consistent with its reported specificity for higher-affinity Aβ aggregates [28].

The current study demonstrates that both SUVr and AUC exhibit negative correlations with the CSF Aβ_42/40_ ratio across most brain regions, with only a few exceptions. In AD patients, CSF Aβ_40_ levels are elevated [29], while CSF Aβ_42_ levels are reduced [30–33], leading to a decreased Aβ_42/40_ ratio [29] compared to non-AD individuals. It is well established that Aβ deposition is more pronounced in cortical and hippocampal regions in human AD patients [34]. Autoradiography findings from this study align with previous reports, confirming that [^18^F]FC119S effectively targets Aβ in these brain regions in vervets. This suggests that radiotracer uptake reflects amyloid accumulation in vervets similar to humans. However, statistical significance was not achieved in the current analysis, maybe due to the limited number of vervet subjects.

Increased CSF pTau181 [35], CSF NfL [36, 37], and an elevated CSF pTau181/Aβ_42_ ratio [38] have been reported previously in human AD patients relative to controls. In the present study, among all CSF biomarkers CSF pTau181, CSF NfL, and CSF pTau181/Aβ_42_ ratio displayed significant correlations with [^18^F]FC119S uptake. Detailed regional analyses revealed significant positive associations between both SUVr and AUC values in the temporal lobe and CSF pTau181 and the CSF pTau181/Aβ42 ratio. In the clinical pathology of AD, amyloid plaques predominantly accumulate in the temporal lobe, particularly the medial temporal lobe—an area critical for memory storage often regarded as the “epicenter” of AD pathology. Extensive plaque deposition in the medial temporal lobe is associated with early memory impairments, making the temporal lobe one of the first affected areas in human AD [39]. The CSF pTau181 biomarker and the CSF pTau181/Aβ_42_ ratio are considered as indicators to detect early pathological changes of human AD progression [40]. Aligned with the regional specificity of AD pathology, our study observed that elevated CSF pTau181 levels and an increased CSF pTau181/Aβ_42_ ratio were associated with significantly higher [^18^F]FC119S uptake in the temporal region of vervets. This finding supports the view that the temporal lobe serves as an early site of amyloid accumulation and associated tau pathology in vervets, mirroring patterns observed in humans. Consequently, the strong association between [^18^F]FC119S uptake and CSF pTau181 as well as the pTau181/Aβ_42_ ratio highlights the potential utility of this tracer for assessing AD progression or age-related changes in vervets.

Additionally, SUVr values showed significant correlations with CSF NfL levels in the anterior cingulate gyrus and parietal lobe. Furthermore, AUC values were significantly associated with CSF NfL in the parietal and occipital lobes. In human AD, the anterior cingulate gyrus is regarded as a key epicenter and among the earliest regions to exhibit pathological changes [41–44]. Amyloid deposition occurs in anterior cingulate gyrus in the initial stages of human AD and the most common phenomenon in parietal and occipital lobes in neurodegenerative diseases like AD [41, 45]. Current study demonstrated that [^18^F]FC119S uptake in anterior cingulate gyrus, parietal and occipital lobes (associated with amyloid deposition) positively correlated with CSF NfL (associated with neurodegeneration) in vervets. As CSF NfL is a direct measure for neurodegeneration, the present association of [^18^F]FC119S uptake with CSF NfL supports its effective usage in the detection of neurodegeneration in vervet monkeys analogous to what is seen in humans [36, 37].

In case of plasma fluid biomarkers, human AD patients exhibit decreased levels of plasma Aβ_40_ and Aβ_42_ compared to cognitively normal individuals [46]. Plasma Aβ_42_/Aβ_40_ ratio is linked to the early stages of AD and is significantly lower in mild cognitively impaired and AD subjects compared to normal humans [46, 47]. Furthermore, plasma pTau181 [48, 49], plasma NfL [50], and plasma pTau181/Aβ_42_ ratio [40, 49] are increased in human AD. While SUVrs with initial 5- and 15- min binning showed no significant correlations with any of the plasma markers, CSF NfL demonstrated significant positive associations in the anterior cingulate (5 min: *r* = 0.63, *p* = 0.006; 15 min: *r* = 0.53, *p* = 0.02), posterior cingulate (5 min: *r* = 0.63, *p* = 0.02; 15 min: *r* = 0.48, *p* = 0.04), hippocampus (5 min: *r* = 0.53, *p* = 0.02; 15 min: *r* = 0.56, *p* = 0.01) and white matter (5 min: *r* = 0.56, *p* = 0.01; 15 min: *r* = 0.51, *p* = 0.03). In the current study, correlations between both SUVrs and AUCs with plasma biomarkers were not statistically significant, limiting the ability to draw definitive conclusions.

This study has a few limitations that warrant consideration. The relatively small sample size may reduce statistical power and limit the generalizability of the findings. Cross-sectional design restricts our ability to assess longitudinal changes or establish causal relationships. The absence of behavioral and cognitive assessments further limits the interpretation of neuroimaging results in the context of functional outcomes. Inclusion of only female vervet monkeys was intended to minimize variability associated with sex-based behavioral and physiological differences; however, this choice may constrain the applicability of the findings to male subjects or mixed-sex populations. Future research will aim to incorporate larger, more diverse cohorts, longitudinal designs, and integrated behavioral measures to enhance the robustness and translational relevance of the findings.

Overall, [^18^F]FC119S showed significant correlations with CSF biomarkers (pTau181, NfL, and pTau181/Aβ_42_ ratio) in cortical regions specifically anterior cingulate gyrus, temporal lobe, parietal lobe, and occipital lobes in vervets. Like humans, [^18^F]FC119S displayed negative associations with Aβ_42/40_ ratio in most of the Aβ-effected brain regions in vervets. These significant associations support the utility of [^18^F]FC119S as an Aβ tracking tracer in vervets, linking its uptake to established CSF biomarkers in AD pathology. Ongoing evaluations are further exploring the association of Aβ PET imaging data and synaptic density and microtubule integrity, within the same cohort to elucidate potential neurodegenerative pathways in aged vervet monkeys.

## Conclusion

The synthesis of [^18^F]FC119S was successfully optimized and fully automated. PET imaging in aged vervet monkeys demonstrates high [^18^F]FC119S uptake in brain regions associated with Aβ deposition, consistent with patterns observed in human PET studies. Like humans, [^18^F]FC119S uptake in vervets showed negative associations with the Aβ_42/40_ ratio across most regions. Among the fluid biomarkers analyzed, significant correlations were identified with CSF pTau181, CSF NfL, and the CSF pTau181/Aβ_42_ ratio. Collectively, these findings suggest that [^18^F]FC119S is a promising PET radiotracer for in vivo Aβ imaging in aged vervet models, supporting its potential for future translational applications in clinical research.

## Supplementary Information

Below is the link to the electronic supplementary material.

Supplementary Material 1

## Data Availability

The datasets used and/or analyzed during the current study are available from the corresponding author on reasonable request.

## Abbreviations

NHP: Non-Human Primates
Aβ: Beta-Amyloid
AD: Alzheimer’s Disease
CSF: Cerebrospinal Fluid
MRI: Magnetic Resonance Imaging
PET: Positron Emission Tomography
nM: Nanomolar
µM: Micromolar
CT: Computed Tomography
HPLC: High-Performance Liquid Chromatography
UV: Ultraviolet
VRC: Vervet Research Colony
IACUC: Institutional Animal Care and Use Committee
NHP: Non-Human Primates
NfL: Neurofilament Light Chain
SUV: Standard Uptake Value
TAC: Time-Activity Curve
SUVr: Standardized Uptake Value Ratio
AUC: Area Under the Curve
