# Supplementary material for: PET imaging utility of a novel Aβ-tracking PET radiotracer, [18F]FC119S in aged vervet monkeys
Source: J Transl Med. 2026 Jan 8;24:42. doi: 10.1186/s12967-025-07642-5 (PMC12784507; doi:10.1186/s12967-025-07642-5)
Supplement: Supplementary file 1 — Supplementary Material 1 [file 12967_2025_7642_MOESM1_ESM.docx]

**Supplementary information**

**PET imaging utility of a novel Aβ-tracking PET radiotracer, [^18^F]FC119S in aged vervet monkeys**

Bhuvanachandra Bhoopal^1^, Brett M Frye^2^, Mack Miller^1^, Avinash Bansode^1^, Krishna K Gollapelli^1^, Richard A Barcus^1^, Samuel N Lockhart^3^, Naresh Damuka^1^, Courtney L Sutphen^2^, Ryan W Fitzgerald,^1^ Jeongchul Kim,^1^ Mark G Baxter,^2^ Matthew J Jorgensen^2^, Suzanne Craft^3^, Thomas C. Register^2^, Christopher T Whitlow^1^, Carol A Shively^2^, Kiran K Solingapuram Sai^1^*

*^1^Department of Radiology; Wake Forest University School of Medicine, Winston-Salem, NC, USA.*

*^2^Department of Pathology, Section on Comparative Medicine, Wake Forest University School of Medicine, Winston-Salem, NC, USA.*

*^3^Department of Internal Medicine, Wake Forest University School of Medicine, Winston-Salem, NC, USA.*

*Corresponding author

Kiran K Solingapuram Sai, PhD

Department of Radiology

Wake Forest University School of Medicine

Winston-Salem

NC 27157

Kiran.solingapuramsai@advocatehealth.org

Ph: 336-716-5630

Table S1. SUVr values for each subject in different regions of brain.

| **Subject ID** | **Anterior**  **Cingulate**  **gyrus** | **Posterior**  **cingulate**  **gyrus** | **Hippocampus** | **Parietal lobe** | **Temporal lobe** | **Frontal lobe** | **Occipital lobe** | **Cortex** | **Gray matter** | **White matter** | **Whole brain** |
| --- | --- | --- | --- | --- | --- | --- | --- | --- | --- | --- | --- |
| **1064** | 1.1779 | 1.0205122 | 1.074703 | 1.2458657 | 1.2260573 | 1.424939292 | 1.02459183 | 1.270424 | 1.2047674 | 1.0575187 | 1.208685 |
| **1068** | 1.12266 | 1.0207793 | 1.082479 | 1.0137643 | 1.1344475 | 1.04950094 | 0.928024593 | 1.041453 | 1.052024 | 0.9098233 | 1.058086 |
| **1069** | 1.026746 | 0.9134807 | 0.887813 | 0.9508518 | 1.162995 | 1.168130067 | 0.871639062 | 1.071892 | 1.0526575 | 0.9640341 | 1.060695 |
| **1097** | 1.138688 | 1.1585246 | 1.118337 | 1.1217747 | 1.0807519 | 1.208322755 | 1.034521528 | 1.121544 | 1.1329201 | 0.949618 | 1.134014 |
| **1112** | 0.882671 | 0.9428457 | 1.019024 | 0.887838 | 0.9700604 | 0.880762088 | 0.993866141 | 0.928666 | 0.9568409 | 0.7673129 | 0.936571 |
| **1123** | 1.097295 | 1.0584619 | 1.027392 | 1.2117384 | 1.1110814 | 1.146024369 | 1.116875012 | 1.14432 | 1.1298966 | 0.8791543 | 1.132938 |
| **1124** | 0.921085 | 0.9438681 | 0.980928 | 1.0118077 | 1.0803493 | 1.033885987 | 0.993498727 | 1.032292 | 1.0273364 | 0.8401681 | 1.010869 |
| **1185** | 0.460475 | 0.3053248 | 0.809454 | 0.4228922 | 1.3293483 | 1.015022966 | 0.636573204 | 0.942815 | 0.9583798 | 1.1035238 | 0.962974 |
| **1233** | 1.178213 | 1.1220537 | 0.973748 | 1.0684516 | 1.0458962 | 1.20160833 | 0.921847407 | 1.086494 | 1.0681988 | 0.9139231 | 1.073127 |
| **1297** | 1.206494 | 1.1276938 | 1.128902 | 1.1976153 | 1.1594233 | 1.188432333 | 1.135125783 | 1.17011 | 1.1692737 | 0.921862 | 1.162705 |
| **1306** | 1.297568 | 1.1507094 | 1.255334 | 1.0980686 | 1.2191963 | 1.045380462 | 0.93265167 | 1.082514 | 1.1031903 | 0.836193 | 1.111203 |
| **1364** | 1.181187 | 1.0958943 | 1.019763 | 1.0309816 | 1.0556139 | 1.161947811 | 0.952455644 | 1.076565 | 1.0645408 | 0.883305 | 1.069536 |
| **1366** | 1.05667 | 1.068444 | 0.972339 | 1.0773039 | 1.0024239 | 1.073336877 | 0.963813399 | 1.037286 | 1.0336798 | 0.8633685 | 1.037936 |
| **1401** | 1.070863 | 1.0655485 | 1.015276 | 1.15876 | 1.1513033 | 1.245332923 | 1.089062688 | 1.170518 | 1.1433184 | 0.9479924 | 1.153299 |
| **1434** | 1.1578 | 1.0575824 | 1.163389 | 1.2328321 | 1.2210869 | 1.235871419 | 1.183746136 | 1.219474 | 1.1906168 | 0.9120805 | 1.204472 |
| **1531** | 1.119039 | 1.0381859 | 0.976482 | 1.0435336 | 1.0893908 | 1.1575802 | 0.970699504 | 1.086821 | 1.0791777 | 1.0886275 | 1.120823 |
| **1656** | 0.775897 | 0.369682 | 1.04424 | 0.5545603 | 1.7006289 | 1.512699904 | 0.65457014 | 1.21881 | 1.1653922 | 1.4022434 | 1.177313 |

Table S2. AUC values for each subject in different regions of brain.

| **Subject ID** | **Anterior**  **Cingulate**  **gyrus** | **Posterior**  **cingulate**  **gyrus** | **Hippocampus** | **Parietal lobe** | **Temporal lobe** | **Frontal lobe** | **Occipital lobe** | **Cortex** | **Gray matter** | **White matter** | **Whole brain** |
| --- | --- | --- | --- | --- | --- | --- | --- | --- | --- | --- | --- |
| **1064** | 7409 | 7312 | 7798 | 10883 | 9470 | 12826 | 8960 | 10801 | 9872 | 8861 | 10081 |
| **1068** | 7678 | 7784 | 8073 | 7352 | 8085 | 8053 | 6908 | 7677 | 7761 | 7992 | 7951 |
| **1069** | 6441 | 6633 | 6452 | 6259 | 7852 | 8027 | 6121 | 7203 | 7261 | 7192 | 7376 |
| **1097** | 9574 | 9853 | 7886 | 8037 | 7758 | 10038 | 7289 | 8455 | 8568 | 9448 | 8953 |
| **1112** | 6685 | 8490 | 7997 | 6949 | 7391 | 8024 | 7267 | 7486 | 7605 | 8509 | 7569 |
| **1123** | 7928 | 8228 | 7077 | 7766 | 7145 | 8280 | 6777 | 7555 | 7696 | 8505 | 8021 |
| **1124** | 7050 | 7521 | 7375 | 6797 | 7522 | 7979 | 6581 | 7302 | 7436 | 7794 | 7406 |
| **1185** | 2485 | 2175 | 5334 | 3371 | 8776 | 5928 | 6643 | 6246 | 6491 | 3966 | 6876 |
| **1233** | 8812 | 9414 | 7884 | 8138 | 7523 | 10254 | 7205 | 8485 | 8397 | 9103 | 8637 |
| **1297** | 8861 | 9389 | 7840 | 8179 | 7700 | 9076 | 7606 | 8206 | 8462 | 9541 | 8594 |
| **1306** | 7931 | 7952 | 8717 | 7403 | 8016 | 6899 | 7103 | 7324 | 7659 | 8778 | 8145 |
| **1364** | 8134 | 8506 | 7622 | 7250 | 7474 | 9071 | 6829 | 7851 | 7830 | 8151 | 8069 |
| **1366** | 7988 | 9144 | 7789 | 8019 | 7473 | 9157 | 7340 | 8113 | 8106 | 8663 | 8298 |
| **1401** | 8051 | 8668 | 7125 | 7475 | 7638 | 9430 | 6982 | 8031 | 8120 | 8516 | 8351 |
| **1434** | 8276 | 8243 | 7810 | 7961 | 8085 | 9241 | 7361 | 8269 | 8251 | 8814 | 8491 |
| **1531** | 7651 | 8085 | 7384 | 7791 | 8252 | 9263 | 7500 | 8355 | 8311 | 8496 | 8629 |
| **1656** | 4334 | 2711 | 7225 | 4249 | 11138 | 9009 | 5751 | 7847 | 7717 | 4964 | 7473 |

Table S3. Correlation coefficients between SUVrs of different regions of brain and age and fluid biomarkers.

|  |  | **Anterior**  **Cingulate**  **gyrus** | **Posterior**  **cingulate**  **gyrus** | **Hippocampus** | **Parietal lobe** | **Temporal lobe** | **Frontal lobe** | **Occipital lobe** | **Cortex** | **Gray matter** | **White matter** | **Whole brain** |
| --- | --- | --- | --- | --- | --- | --- | --- | --- | --- | --- | --- | --- |
| **Age** | **r value** | 0.2094 | 0.3998 | 0.1476 | 0.3914 | -0.4412 | -0.3425 | 0.479 | -0.09404 | -0.02281 | -0.6242 | -0.1009 |
|  | **p value** | 0.4199 | 0.1118 | 0.5718 | 0.1203 | 0.0763 | 0.1784 | 0.0517 | 0.7196 | 0.9308 | 0.0074 | 0.7 |
| **CSF**  **Aβ_40_** | **r value** | 0.09689 | 0.1917 | 0.07803 | 0.2977 | -0.1969 | 0.1088 | 0.3687 | 0.2159 | 0.2258 | -0.1064 | 0.2095 |
|  | **p value** | 0.7114 | 0.461 | 0.766 | 0.2459 | 0.4488 | 0.6778 | 0.1453 | 0.4053 | 0.3835 | 0.6844 | 0.4196 |
| **CSF**  **Aβ_42_** | **r value** | 0.01313 | 0.1049 | 0.106 | 0.2445 | -0.09347 | 0.0694 | 0.3823 | 0.217 | 0.2397 | -0.1029 | 0.2195 |
|  | **p value** | 0.9601 | 0.6888 | 0.6856 | 0.3442 | 0.7212 | 0.7913 | 0.1299 | 0.4028 | 0.3541 | 0.6943 | 0.3973 |
| **CSF**  **Aβ_42/40_ ratio** | **r value** | -0.2016 | -0.1365 | 0.003747 | 0.01387 | 0.1165 | -0.01955 | 0.2236 | 0.1125 | 0.1408 | -0.02107 | 0.1178 |
|  | **p value** | 0.4378 | 0.6013 | 0.9886 | 0.9579 | 0.656 | 0.9406 | 0.3883 | 0.6672 | 0.5899 | 0.936 | 0.6525 |
| **CSF**  **pTau181** | **r value** | -0.02871 | -0.266 | 0.4073 | -0.2669 | 0.5388 | 0.2298 | -0.3675 | 0.1537 | 0.1567 | 0.284 | 0.1348 |
|  | **p value** | 0.9129 | 0.3021 | 0.1046 | 0.3004 | 0.0256 | 0.3749 | 0.1467 | 0.5559 | 0.5481 | 0.2692 | 0.606 |
| **CSF**  **NfL** | **r value** | 0.5106 | 0.4412 | 0.481 | 0.5664 | -0.1275 | 0.05611 | 0.4493 | 0.3453 | 0.3952 | -0.325 | 0.3397 |
|  | **p value** | 0.0362 | 0.0762 | 0.0507 | 0.0178 | 0.6259 | 0.8306 | 0.0704 | 0.1746 | 0.1164 | 0.2031 | 0.1822 |
| **CSF**  **pTau181/Aβ_42_** | **r value** | -0.029 | -0.2735 | 0.3951 | -0.3139 | 0.5494 | 0.1773 | -0.4232 | 0.08789 | 0.09754 | 0.29 | 0.08978 |
|  | **p value** | 0.912 | 0.2882 | 0.1165 | 0.2199 | 0.0224 | 0.496 | 0.0905 | 0.7373 | 0.7096 | 0.2588 | 0.7318 |
| **Plasma**  **Aβ_40_** | **r value** | 0.562 | 0.5334 | 0.4427 | 0.5314 | -0.1787 | 0.1407 | 0.4575 | 0.3343 | 0.4066 | -0.228 | 0.3746 |
|  | **p value** | 0.0189 | 0.0275 | 0.0751 | 0.0282 | 0.4927 | 0.5901 | 0.0648 | 0.1897 | 0.1053 | 0.3788 | 0.1385 |
| **Plasma**  **Aβ_42_** | **r value** | 0.5079 | 0.3872 | 0.6091 | 0.3121 | -0.03033 | -0.08653 | 0.1255 | 0.1025 | 0.1786 | -0.2547 | 0.1866 |
|  | **p value** | 0.0446 | 0.1384 | 0.0123 | 0.2393 | 0.9112 | 0.75 | 0.6434 | 0.7057 | 0.5081 | 0.3412 | 0.489 |
| **Plasma**  **Aβ_42/40_ ratio** | **r value** | 0.3479 | 0.2321 | 0.4346 | 0.1468 | -0.00518 | -0.1403 | -0.04694 | -0.0265 | 0.01298 | -0.2035 | 0.03827 |
|  | **p value** | 0.1867 | 0.3871 | 0.0925 | 0.5874 | 0.9848 | 0.6042 | 0.8629 | 0.9224 | 0.962 | 0.4496 | 0.8881 |
| **Plasma**  **pTau181** | **r value** | -0.5034 | -0.3461 | -0.629 | -0.4039 | -0.1301 | -0.3152 | -0.4213 | -0.5042 | -0.5788 | -0.04625 | -0.5843 |
|  | **p value** | 0.0394 | 0.1736 | 0.0068 | 0.1078 | 0.6187 | 0.2178 | 0.0922 | 0.039 | 0.0149 | 0.8601 | 0.0138 |
| **Plasma NfL** | **r value** | 0.1401 | 0.167 | 0.1092 | 0.2383 | -0.0711 | 0.04381 | 0.3111 | 0.1816 | 0.2324 | -0.11 | 0.1703 |
|  | **p value** | 0.5917 | 0.5218 | 0.6765 | 0.3569 | 0.7863 | 0.8674 | 0.2241 | 0.4854 | 0.3694 | 0.6742 | 0.5134 |
| **Plasma pTau181/Aβ_42_** | **r value** | -0.7566 | -0.5978 | -0.6764 | -0.6084 | 0.05152 | -0.376 | -0.5291 | -0.565 | -0.619 | 0.08539 | -0.6139 |
|  | **p value** | 0.0007 | 0.0145 | 0.004 | 0.0124 | 0.8497 | 0.1512 | 0.0351 | 0.0226 | 0.0106 | 0.7532 | 0.0114 |

Table S4. Correlation coefficients between AUCs of different regions of brain and age and fluid biomarkers.

|  |  | **Anterior**  **Cingulate**  **gyrus** | **Posterior**  **cingulate**  **gyrus** | **Hippocampus** | **Parietal lobe** | **Temporal lobe** | **Frontal lobe** | **Occipital lobe** | **Cortex** | **Gray matter** | **White matter** | **Whole brain** |
| --- | --- | --- | --- | --- | --- | --- | --- | --- | --- | --- | --- | --- |
| **Age** | **r value** | 0.2945 | 0.4072 | 0.06558 | 0.2569 | -0.5772 | -0.08598 | 0.2106 | -0.07388 | -0.00323 | 0.3601 | 0.0677 |
|  | **p value** | 0.2511 | 0.1047 | 0.8025 | 0.3195 | 0.0153 | 0.7428 | 0.4172 | 0.7781 | 0.9902 | 0.1556 | 0.7963 |
| **CSF**  **Aβ_40_** | **r value** | 0.2473 | 0.19 | -0.03196 | 0.2985 | -0.1389 | 0.3201 | 0.3108 | 0.2929 | 0.2895 | 0.2211 | 0.337 |
|  | **p value** | 0.3385 | 0.4651 | 0.9031 | 0.2446 | 0.595 | 0.2104 | 0.2246 | 0.254 | 0.2597 | 0.3937 | 0.1859 |
| **CSF**  **Aβ_42_** | **r value** | 0.1725 | 0.1156 | -0.08654 | 0.1662 | -0.1264 | 0.1641 | 0.184 | 0.1395 | 0.151 | 0.1519 | 0.1893 |
|  | **p value** | 0.508 | 0.6587 | 0.7412 | 0.5237 | 0.6288 | 0.529 | 0.4797 | 0.5933 | 0.563 | 0.5606 | 0.4668 |
| **CSF**  **Aβ_42/40_ ratio** | **r value** | -0.07678 | -0.1049 | -0.2694 | -0.1524 | -0.05839 | -0.1637 | -0.1065 | -0.1866 | -0.1639 | -0.07211 | -0.1545 |
|  | **p value** | 0.7696 | 0.6887 | 0.2957 | 0.5593 | 0.8238 | 0.5302 | 0.6841 | 0.4732 | 0.5296 | 0.7833 | 0.5538 |
| **CSF**  **pTau181** | **r value** | -0.2702 | -0.3511 | 0.2831 | -0.1872 | 0.5271 | -0.1014 | -0.1808 | -0.01056 | -0.04747 | -0.2251 | -0.09399 |
|  | **p value** | 0.2943 | 0.167 | 0.2709 | 0.4719 | 0.0297 | 0.6987 | 0.4874 | 0.9679 | 0.8564 | 0.385 | 0.7197 |
| **CSF**  **NfL** | **r value** | 0.3342 | 0.3223 | 0.3844 | 0.5847 | -0.1136 | 0.2421 | 0.504 | 0.3925 | 0.4238 | 0.4617 | 0.4497 |
|  | **p value** | 0.1898 | 0.2071 | 0.1276 | 0.0137 | 0.6643 | 0.3492 | 0.0391 | 0.1191 | 0.09 | 0.0621 | 0.0701 |
| **CSF**  **pTau181/Aβ_42_** | **r value** | -0.2778 | -0.3541 | 0.2985 | -0.2693 | 0.5109 | -0.2035 | -0.2758 | -0.1151 | -0.1353 | -0.2545 | -0.1827 |
|  | **p value** | 0.2803 | 0.1632 | 0.2445 | 0.2958 | 0.0361 | 0.4334 | 0.2839 | 0.6601 | 0.6047 | 0.3242 | 0.4828 |
| **Plasma**  **Aβ_40_** | **r value** | 0.5847 | 0.5198 | 0.4487 | 0.4134 | -0.2878 | 0.2402 | 0.1392 | 0.2212 | 0.3352 | 0.5525 | 0.3624 |
|  | **p value** | 0.0137 | 0.0325 | 0.0708 | 0.0991 | 0.2627 | 0.353 | 0.5941 | 0.3937 | 0.1885 | 0.0215 | 0.1528 |
| **Plasma**  **Aβ_42_** | **r value** | 0.2799 | 0.2273 | 0.5569 | 0.2762 | -0.05886 | -0.07591 | 0.2007 | 0.08102 | 0.142 | 0.3352 | 0.2294 |
|  | **p value** | 0.2937 | 0.3971 | 0.025 | 0.3004 | 0.8286 | 0.7799 | 0.4561 | 0.7655 | 0.6 | 0.2043 | 0.3928 |
| **Plasma**  **Aβ_42/40_ ratio** | **r value** | 0.0925 | 0.06685 | 0.4281 | 0.1708 | 0.02554 | -0.1271 | 0.1863 | 0.03817 | 0.05065 | 0.1681 | 0.1333 |
|  | **p value** | 0.7333 | 0.8057 | 0.0981 | 0.527 | 0.9252 | 0.639 | 0.4896 | 0.8884 | 0.8522 | 0.5336 | 0.6227 |
| **Plasma**  **pTau181** | **r value** | -0.3867 | -0.2788 | -0.4205 | -0.265 | -0.07399 | -0.1919 | -0.03169 | -0.2232 | -0.2958 | -0.3691 | -0.2752 |
|  | **p value** | 0.1252 | 0.2785 | 0.0928 | 0.304 | 0.7778 | 0.4607 | 0.9039 | 0.3893 | 0.2491 | 0.1449 | 0.2851 |
| **Plasma NfL** | **r value** | 0.2018 | 0.2261 | 0.0415 | 0.2 | -0.1496 | 0.1137 | 0.2598 | 0.1281 | 0.2133 | 0.2346 | 0.1855 |
|  | **p value** | 0.4373 | 0.3828 | 0.8743 | 0.4416 | 0.5665 | 0.6639 | 0.3139 | 0.6241 | 0.411 | 0.3647 | 0.4761 |
| **Plasma pTau181/Aβ_42_** | **r value** | -0.6669 | -0.5484 | -0.6692 | -0.5127 | 0.04879 | -0.4509 | -0.1364 | -0.4331 | -0.5157 | -0.6349 | -0.4722 |
|  | **p value** | 0.0048 | 0.0278 | 0.0046 | 0.0423 | 0.8576 | 0.0796 | 0.6146 | 0.0938 | 0.0409 | 0.0082 | 0.0648 |
